# Spatio-Semantic Graphs From Picture Description: Applications to Detection of Cognitive Impairment

**DOI:** 10.3389/fneur.2021.795374

**Published:** 2021-12-09

**Authors:** Pranav S. Ambadi, Kristin Basche, Rebecca L. Koscik, Visar Berisha, Julie M. Liss, Kimberly D. Mueller

**Affiliations:** ^1^College of Health Solutions, Arizona State University, Tempe, AZ, United States; ^2^Division of Geriatrics, School of Medicine and Public Health, University of Wisconsin-Madison, Madison, WI, United States; ^3^Wisconsin Alzheimer's Institute, School of Medicine and Public Health, University of Wisconsin-Madison, Madison, WI, United States; ^4^Department of Communication Sciences and Disorders, University of Wisconsin-Madison, Madison, WI, United States

**Keywords:** Alzheimer's disease, dementia, speech biomarkers, cognition, semantic analysis, cookie theft, graph theory

## Abstract

Clinical assessments often use complex picture description tasks to elicit natural speech patterns and magnify changes occurring in brain regions implicated in Alzheimer's disease and dementia. As The Cookie Theft picture description task is used in the largest Alzheimer's disease and dementia cohort studies available, we aimed to create algorithms that could characterize the visual narrative path a participant takes in describing what is happening in this image. We proposed spatio-semantic graphs, models based on graph theory that transform the participants' narratives into graphs that retain semantic order and encode the visuospatial information between content units in the image. The resulting graphs differ between Cognitively Impaired and Unimpaired participants in several important ways. Cognitively Impaired participants consistently scored higher on features that are heavily associated with symptoms of cognitive decline, including repetition, evidence of short-term memory lapses, and generally disorganized narrative descriptions, while Cognitively Unimpaired participants produced more efficient narrative paths. These results provide evidence that spatio-semantic graph analysis of these tasks can generate important insights into a participant's cognitive performance that cannot be generated from semantic analysis alone.

## Introduction

Asking patients to describe a complex picture is a mainstay of clinical assessment tasks in aphasia, and increasingly so in the context of cognitive decline and dementia (1). This task is straight-forward to elicit, and its successful completion requires the ability to scan the scene, retrieve and sequence the relevant semantic symbols, and draw inferences about relationships and causation among the objects in the scene (2). The complexity of the cognitive-linguistic processing required for an accurate and comprehensive description makes the task an ideal candidate to magnify early and mild changes associated with medial temporal lobe and frontal lobe pathology (3).

The Cookie Theft picture description task from the Boston Diagnostic Aphasia Examination (4) is the most commonly elicited task, both clinically and in research and across a broad range of cognitive-linguistic conditions (2). The black and white line drawing portrays a kitchen scene, in which a mother is absentmindedly drying dishes at the sink while the running water overflows onto the floor. Behind her is her son, who stands atop a wobbly stool stealing cookies from a cookie jar for himself and for his sister, who holds out her hand in anticipation. The curtained window above the sink opens to a yard scene. Typically, transcripts of the spoken picture descriptions are coded by hand by trained individuals to tag parts of speech, content information units (CIUs) (content units, semantic relevance), empty speech, repetitions, among others; as well as acoustic measures extracted from speech recordings (1, 5–7). These data have been used to detect preclinical changes in cognitive-linguistics and differentiate among dementia etiologies such as Alzheimer's (AD), frontotemporal dementia (FTD), dementia due to Parkinson's disease (PD) and dementia with Lewy bodies (DLB) (8–10).

While there is nothing particularly special about The Cookie Theft picture itself—and indeed it has been criticized and even revised for being outdated and culturally non-inclusive (11, 12)—the original picture from the BDAE enjoys the status of having been used to elicit spontaneous speech data in the largest Alzheimer's disease and dementia cohort studies to date (13–15). As such, these picture description data are incomparable in their potential, in joint consideration with other biomarkers and data, to provide for increasingly earlier detection of pathological changes in AD and other dementias. Preclinical detection is essential for the development of disease altering interventions (16).

While the promise of cognitive-linguistic and acoustic metrics in the analysis of picture descriptions is high, these approaches leave important information on the table. In particular, they underspecify the ways in which the patient navigates the visual scene to “describe everything that is happening in the picture,” per the task instructions. Describing a picture has been shown to invoke the expected cortical pathways underlying semantic retrieval and production for the objects in the picture (anterior temporal lobe, inferior frontal gyrus, and sensorimotor cortices), but also to pathways linking aspects of the parietal lobe with posterior cortical circuits activated for visual processing of a picture (3). There is a growing body of evidence that the parietal lobe is among the earliest sites for neurodegenerative AD change; changes in visuospatial abilities may differentiate AD from other dementias (17, 18).

In the current study, we sought to develop algorithms that would allow us to characterize the ways in which participants navigate the visual scene using the large set of The Cookie Theft transcripts in the combined Wisconsin Registry for Alzheimer's Prevention (WRAP) + DementiaBank databases. We conjectured that by tracking the spatial movement paths from semantic object to semantic object, the results would reflect a culmination of visuospatial, attentional, and organizational capabilities. To that end, we apply graph theory and introduce the concept of *spatio-semantic graphs*—mathematical models that encode the sequential listing of content units in a transcript *and* their relative spatial position in The Cookie Theft image. Previous studies have shown the value of analyzing the lexical sequence *via* visual analyses and automatic speech recognition on recorded Cookie Theft descriptions to classify participants as AD or as healthy (19), and *via* natural language processing of dream reports to objectively differentiate normal and dysfunctional flows of thought (20). Along this line of research, we introduce this new graph-based representation with the aim of generating mechanistic and interpretable features capable of sensitively capturing early and emerging cognitive decline. We explore the distribution of these features on existing large-scale corpora to determine if they differ between clinical and control groups in ways that match performance expectations. By restricting our analysis to the transcripts alone to generate these graphs, we unlock additional value from the large amount of data already available to researchers in existing corpora.

## Materials and Methods

### Data

The data for this study accessed 1,058 audio recordings from the WRAP database and 291 audio recordings from the DementiaBank (DB) Pitt Corpus database. WRAP is a longitudinal, observational cohort of individuals in midlife, enriched for parental history of AD. WRAP began in 2001; participants attend study visits every 2 years in which they provide detailed health and lifestyle data, as well as undergo comprehensive neuropsychological testing [see (14) for complete description of WRAP]. Speech sample collection including Cookie Theft picture descriptions began in 2012. The Pitt Corpus from DementiaBank (https://dementia.talkbank.org) consists of audio-recorded data collected as part of a larger protocol administered by the Alzheimer and Related Dementias Study at the University of Pittsburgh School of Medicine (13). All data used in this study were transcripts from the first available audio recordings of The Cookie Theft picture description task only. Control data from DB (*n* = 99 participants) were combined with Cognitively Unimpaired-Stable participant data from WRAP [*n* = 836; (21)]; participants with AD from DB (*n* = 193) were combined with MCI participants from WRAP (*n* = 26). The combined dataset includes four possible diagnoses: Cognitively Unimpaired-Stable (CUS) (935), Cognitively Unimpaired-Declining (CUD) (181 from WRAP), Impaired but not MCI (14 from WRAP), and MCI/Dementia (219). Further participant characteristics, including average age, years of education, and PACC3 scores are included in the Supplementary Material.

Content-information-units (CIUs) used in this study were adapted from Croisile et al. (22), further defined by our group in Mueller et al. (6), and include a total of 23 Subjects, Objects, and Actions/Facts (see Supplementary Material).

### Constructing Spatio-Semantic Graphs From Transcripts

**Figure 1:**
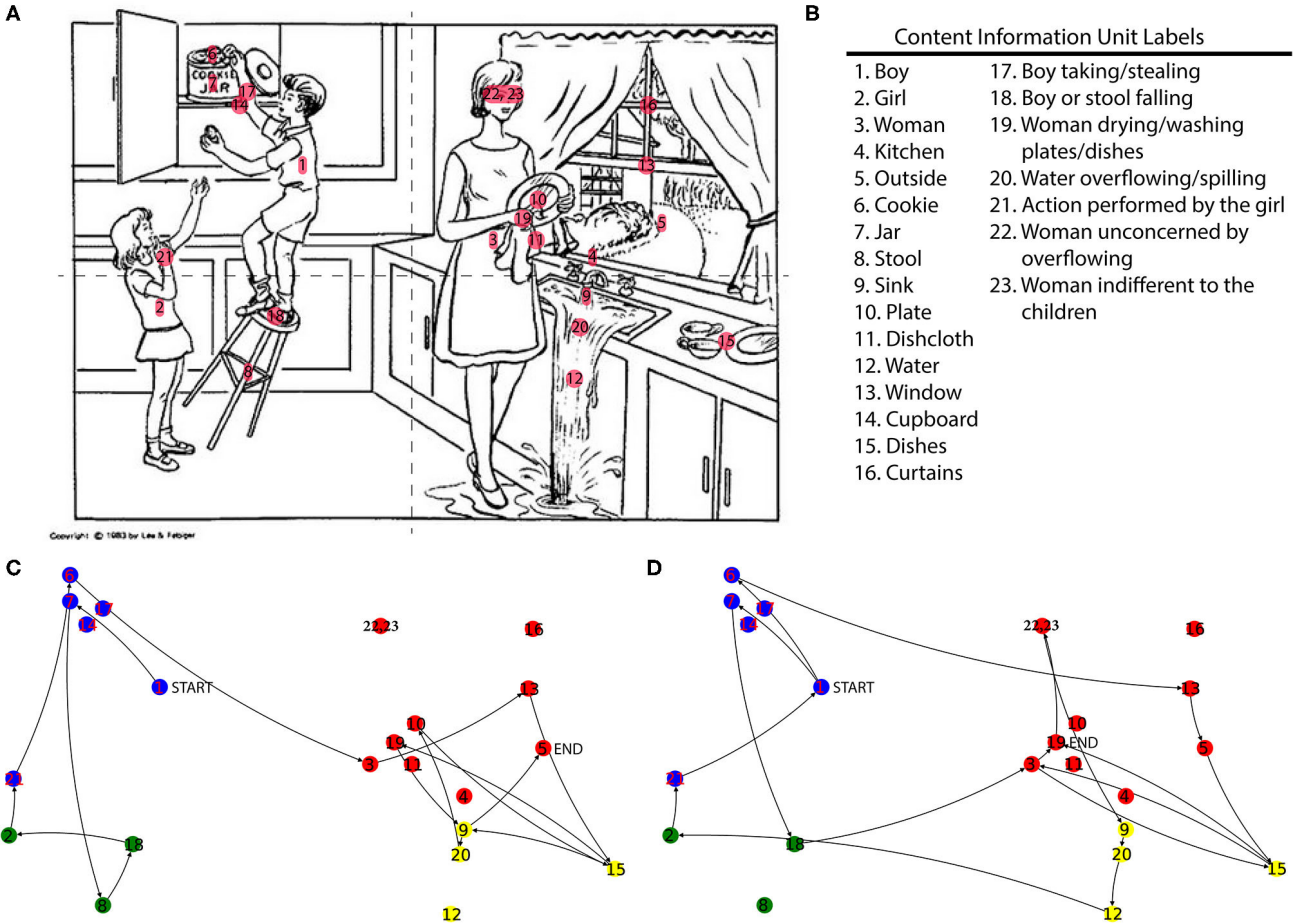
**(A)** The image used in the cookie theft picture description task overlaid with the CIUs at their approximate assigned coordinate locations. Dotted lines indicate the quadrant splits in the image. **(B)** Definitions for the CIU labels. **(C)** Examples of a Healthy Control participant's and **(D)** an AD participant's descriptions transformed into spatio-semantic graphs. Nodes are labeled the same as in panel A and are colored according to quadrants in which nodes fall. Starting and ending nodes are labeled to the right of the corresponding nodes for both participants. While both participants reach the same number of unique nodes mentioned, the AD participant's description has inefficient pathing with several repeats, cross quadrant transitions, and a larger total path distance traveled [(4), Used with Permission].

All participant transcripts were processed in Python. The 23 CIUs were manually assigned *(x, y)* coordinate pairs on a pixel scale on a picture of The Cookie Theft (the copy used was 546 × 290 pixels). Figures 1A,B show a schematic of the approximate relative positions (and the descriptions) of the assigned CIUs overlaid on The Cookie Theft image.

Transcripts from the WRAP and DB databases already have CIUs manually labeled, and these labels and their order of occurrence in the transcript were extracted and automatically encoded with the corresponding coordinate pairs. Next, the NetworkX package was used to transform the CIUs with their corresponding features, including coordinate pairs, and orderings into a set of nodes and edges that can be analyzed and visualized as a graph (23). The graph nodes represent the 23 CIUs in the image and the graph edges encode the order in which CIUs were mentioned in the transcript by the participant and the relative spatial location between two connected CIUs (*via* a Euclidean distance between two nodes connected by an edge). Additionally, each CIU was attributed a quadrant of the picture. Quadrant information was also processed in NetworkX to analyze and visualize participants' transitions between and within quadrants of the picture. In this representation, the nodes in the graph represent the quadrants of the image and the edges represent how the participant is moving across different quadrants as they describe the picture. Examples of transformed participant transcripts as graphs are also shown in Figures 1C,D.

### Extracting Features From Spatio-Semantic Graphs

After transforming the participant transcripts into nodes and edges, several features from the graph were calculated. These features were extracted from the graph representation and the relative spatial position of the CIUs in the image. Additionally, metrics based on quadrant transitions were calculated from participants' transitions within and between these collective nodes. The features, their calculation, and their interpretation are described in Table 1.

**Table 1:** Feature descriptions.

**Features**	**Calculation**	**Interpretation**
Avg. *X*	Average *x* position of all mentioned nodes	The average horizontal position across all CIUs mentioned by the participants. (0,0) is the top left corner of the cookie theft image. Repeated CIUs are counted.
St. Dev. *X*	Standard deviation of *x* positions of all mentioned nodes	This reflects how widely spread the participants' mentioned CIUs are across the horizontal space. A smaller standard deviation is a less disperse narrative, and vice versa.
Avg. *Y*	Average *y* position of all mentioned nodes	The average vertical position across all CIUs mentioned by the participants. (0,0) is the top left corner of the cookie theft image. Repeated CIUs are counted.
St. Dev. *Y*	Standard deviation of *y* positions of all mentioned nodes	This reflects how widely spread the participants' mentioned CIUs are across the vertical space. A smaller standard deviation is a less disperse narrative, and vice versa.
Total path distance	Sum of all edge lengths	The average total distance in pixels covered by each participant's path between all mentioned CIUs. When normalizing for unique nodes, a higher total path distance may indicate a less efficient path.
Total path/unique nodes	The total path distance divided by the number of unique nodes	The average distance between any two mentioned CIUs across all participants' narratives.
Self cycles	The number of consecutive occurrences of a node in a transcript	The number of times participants mention the same CIU consecutively. A higher number of self cycles may indicate fixation or repetition.
Cycles	All repeated mentions of nodes, consecutive and nonconsecutive	The number of times participants repeat a CIU, including consecutively (self cycles) and non-consecutively. A higher number of cycles may indicate short term memory lapses or fixation.
Nodes	The number of nodes mentioned, including repeats	The number of times participants mention the CIUs in their narrative, including repeat mentions.
Unique nodes	Total number of nodes mentioned, ignoring repeats	How many of the 23 CIUs the participants mentioned.
Self cycles (quadrants)	The total number of edges connecting nodes within the same quadrant	The number of times a participant moves between two CIUs within the same quadrant.
Cross ratio (quadrants)	The number of edges connecting nodes in different quadrants/number of edges connecting nodes in the same quadrant [self cycles (quadrants)]	The number of times participants move between two CIUs in two different quadrants divided by the number of times participants move between two CIUs in the same quadrant. A higher ratio may indicate a more sporadic or unfocused narrative.

*All calculated and analyzed features are listed in this table, along with their method of calculation and interpretations in the context of cognitive performance*.

### Statistical Analysis

For the statistical analysis, two sets of ANCOVA models were used to determine whether there exist group-level differences in the features in Table 1 between individuals who are cognitively impaired and cognitively unimpaired and whether these differences occur at early stages of cognitive impairment.

For the first ANCOVA, the CUS and CUD diagnosis groups were combined into the Cognitively Unimpaired level (*n* = 1,116) of the diagnosis independent variable, and the Impaired but not MCI and MCI/Dementia diagnosis groups were combined into the Cognitively Impaired level (*n* = 233). For the second set, CUS and CUD were used as the two levels of the diagnosis independent variable while Impaired but not MCI and MCI/Dementia groups were omitted. The purpose of the second analysis was to determine whether the spatio-semantic graph features capture pre-clinical changes. Each individual feature was used in its own ANCOVA model as the dependent variable, with demographic data (Age, Education, and Gender) as well as the Unique Nodes variable as covariates. We adjust for Unique Nodes, which is a proxy for the number of content units in the transcript, as it was expected to vary between the Cognitively Unimpaired and Cognitively Impaired groups. To verify this, an initial one-way ANCOVA was performed comparing how these two participant groups differed in the number of Unique Nodes mentioned, while controlling for age, education, and gender. The data for all ANCOVA models were checked for homogeneity of variance (or homoscedasticity) using Levene's test. A significant result indicates that the null hypothesis that the diagnosis groups have equal population variances should be rejected. This violation of one of the assumptions to run an ANCOVA can lead to a decrease in the power of the test (24). In the following analyses, our aim is to evaluate whether the remaining features differ in distribution when compared across the diagnosis groups.

## Results

The sections that follow list the results of the ANCOVAs for the two group comparisons of interest: Cognitively Unimpaired vs. Cognitively Impaired and CUS vs. CUD.

### Cognitively Unimpaired vs. Cognitively Impaired

**Figure 2:**
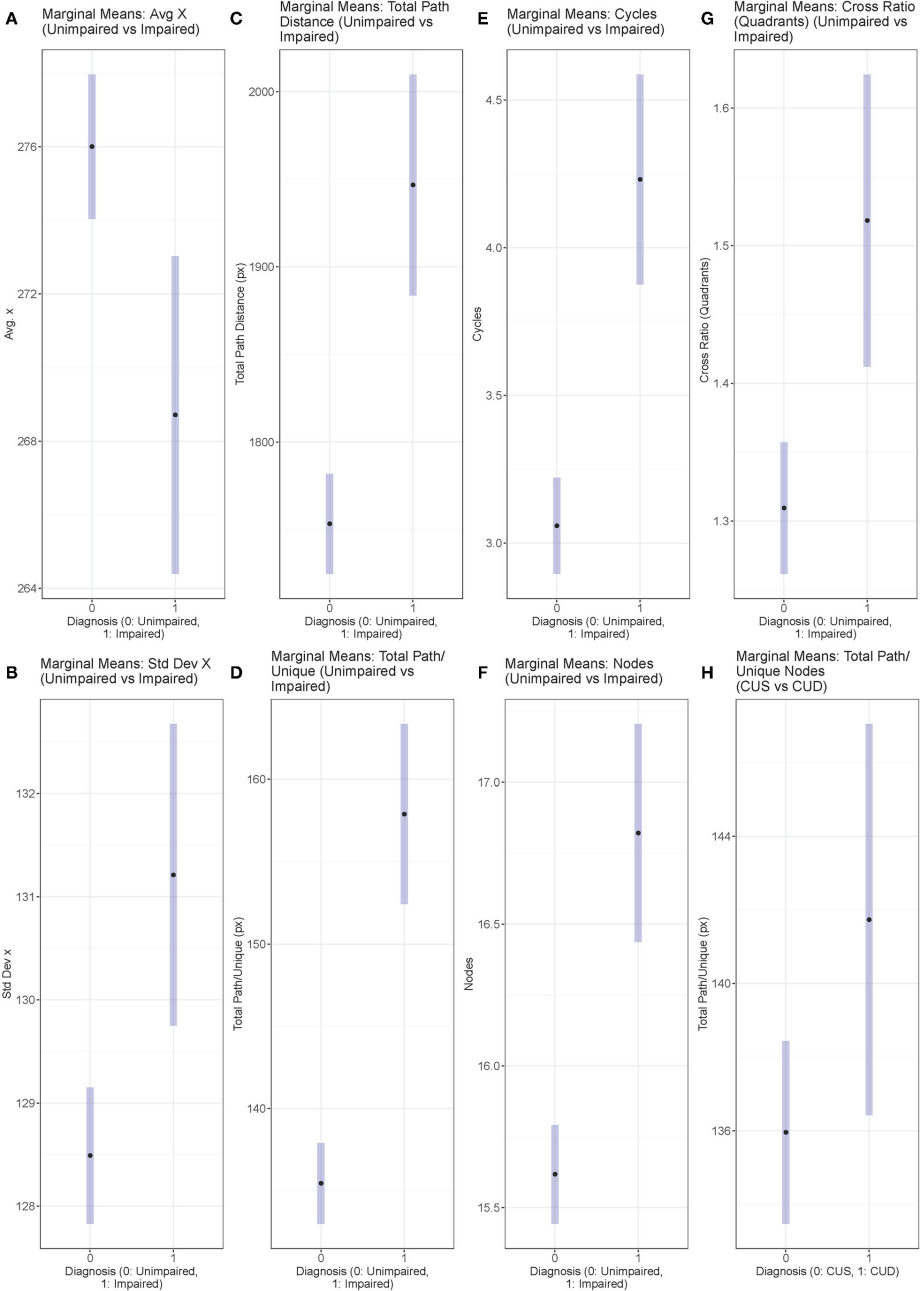
Marginal Means Plots displaying the marginal means for all features with significant results from both sets of ANCOVAs **(A–G)** Unimpaired vs. Impaired, and **(H)** CUS vs. CUD. Marginal means are estimated using model parameters, holding Age, Education, Gender, and Unique Nodes constant.

The top section of Table 2 contains the results for the ANCOVAs performed with the Cognitively Unimpaired and Cognitively Impaired groups as levels of the independent variable diagnosis, and Figure 2 contains plots depicting the Marginal Means of each ANCOVA with a significant result. There was a significant difference in the mean number of Unique Nodes mentioned after adjusting for covariates and the model passed Levene's test of homoscedasticity, so Unique Nodes was also used as a covariate for all other models.

**Table 2:** ANCOVA models for features against two binary comparisons.

**Features**	***F***	***p***	**Marginal mean (95% CI): cognitively unimpaired** **(***n*** = 1,116)**	**Marginal mean (95% CI): cognitively impaired** **(***n*** = 233)**
Avg. *x*	*F*_(1,1288)_ = 8.8480	0.0030	276 (274, 278)	269 (264, 273)
St. Dev. *x*	*F*_(1,1284)_ = 10.8191	0.0010	128 (128, 129)	131 (130, 133)
Avg. *y*	*F*_(1,1924)_ = 1.2897	0.2563	173 (172, 174)	174 (172, 176)
St. Dev. *y*	*F*_(1,1286)_ = 0.0420	0.8376	70.3 (69.8, 70.8)	70.4 (69.3, 71.5)
Total path distance	*F*_(1,1303)_ = 29.3321	<0.001	1,753 (1,725, 1,782)	1,947 (1,884, 2,010)
Total path/unique nodes	*F*_(1,1306)_ = 52.9473	<0.001	135 (133, 138)	158 (152, 163)
Self cycles	*F*_(1,1289)_ = 1.6172	0.2037	0.476 (0.429, 0.523)	0.550 (0.448, 0.653)
Cycles	*F*_(1,1297)_ = 33.8516	<0.001	3.06 (2.90, 3.22)	4.23 (3.88, 4.59)
Nodes	*F*_(1,1289)_ = 30.7300	<0.001	15.6 (15.4, 15.8)	16.8 (16.4, 17.2)
Self cycles (Quadrants)	*F*_(1,1299)_ = 0.8310	0.3621	6.74 (6.60, 6.87)	6.89 (6.59, 7.19)
Cross ratio (Quadrants)	*F*_(1,1294)_ = 12.1103	<0.001	1.31 (1.26, 1.36)	1.52 (1.41, 1.62)
Unique nodes^*^	*F*_(1,1294)_ = 74.7424	<0.001	13.1 (12.9, 13.3)	11 (10.5, 11.4)
**Features**	***F***	***p***	**Marginal mean (95% CI): cognitively unimpaired stable** **(***n*** = 935)**	**Marginal mean (95% CI): cognitively unimpaired declining** **(***n*** = 181)**
Avg. *x*	*F*_(1,1063)_ = 0.0506	0.8220	276 (274, 278)	277 (272, 281)
St. Dev. *x*	*F*_(1,1061)_ = 0.4390	0.5078	129 (128, 130)	130 (128, 131)
Avg. *y*	*F*_(1,1066)_ = 0.0856	0.7699	172 (171, 173)	172 (170, 174)
St. Dev. *y*	*F*_(1,1062)_ = 2.8988	0.0889	70.3 (69.8, 70.8)	71.3 (70.3, 72.4)
Total path distance	*F*_(1,1077)_ = 0.9842	0.3214	1,797 (1,767, 1,827)	1,832 (1,768, 1,897)
Total path/unique nodes	*F*_(1,1084)_ = 3.8398	0.0503	136 (133, 138)	142 (136, 147)
Self cycles	*F*_(1,1062)_ = 0.5060	0.4770	0.495 (0.444, 0.546)	0.452 (0.343, 0.561)
Cycles	*F*_(1,1068)_ = 0.1707	0.6796	3.19 (3.02, 3.35)	3.10 (2.74, 3.47)
Nodes	*F*_(1,1065)_ = 0.1265	0.7222	16.1 (15.9, 16.3)	16.0 (15.6, 16.4)
Self cycles (Quadrants)	*F*_(1,1073)_ = 0.0071	0.9328	6.97 (6.82, 7.11)	6.95 (6.63, 7.27)
Cross ratio (Quadrants)	*F*_(1,1072)_ = 0.0896	0.7647	1.32 (1.27, 1.37)	1.34 (1.23, 1.44)
Unique nodes^*^	*F*_(1,1065)_ = 2.3874	0.1226	13.2 (13.0, 13.4)	12.8 (12.4, 13.3)

*The results from the two sets of ANCOVAs are displayed. The top section reports the ANCOVAs comparing the two collective diagnosis groups, Cognitive Unimpaired (consisting of CUS and CUD) and Cognitive Impaired (Impaired but not MCI, and MCI/Dementia). The bottom section reports ANCOVAs comparing the two Cognitive Unimpaired diagnoses, CUS and CUD. Each feature is the dependent variable in its corresponding model and demographic data (Age, Education, and Gender) as well as Unique Nodes are covariates. All features with dark yellow highlights had p values under 0.05 while those with light yellow highlights had p values close to 0.05*.

* *These models were performed with Unique Nodes as the dependent variable instead of as a covariate with all other covariates remaining, and were performed to verify that Unique Nodes was a useful metric to adjust for in all other models*.

Six dependent variables achieved significance at *p* < 0.05: Average *x* and Standard Deviation of *x*, Total Path Distance/Unique Nodes, Cross Ratio (quadrants), Cycles, Total Path Distance. Levene's test revealed unequal variances for all but the Average *x* feature.

Comparing the marginal means for these features showed that the Cognitively Impaired group had a lower Average *x* position of mentioned nodes (more left aligned) and higher Standard Deviation of *x* compared to the Cognitively Unimpaired group, as well as a higher Total Path Distance/Unique Nodes, Total Path Distance, Cross Ratio (quadrants), Number of Cycles, and Number of nodes mentioned.

### Cognitively Unimpaired-Stable vs. Cognitively Unimpaired-Declining

The bottom section of Table 2 contains the results for the ANCOVAs performed with the Cognitively Unimpaired-Stable and Cognitively Unimpaired-Declining diagnoses as levels of the independent variable diagnosis. As with the first set of models, Unique Nodes was used as a dependent variable in an ANCOVA to verify if there was a significant difference. There was no significant difference found, however we still chose to use Unique Nodes as a covariate for the other models run in this group comparison to maintain consistency with the previous set of models in controlling for this variable. While no features achieved *p*-values less than the threshold value of 0.05, the Total Path Distance/Unique Nodes feature had a *p*-value close to this threshold value. The marginal means show that the Cognitively Unimpaired-Declining group had a higher Total Path Distance/Unique Nodes. Additionally, Levene's test failed to reject the null hypothesis that the variances are equal for this feature.

## Discussion

The ability to identify pre-clinical cognitive changes is essential to the development of interventions that halt or slow irreversible neurodegeneration. The Cookie Theft picture description task has been widely studied for its ability to elicit symptoms associated with early dementia and Alzheimer's disease (6, 25). In this investigation, we developed an approach to extract novel additional information from transcriptions of The Cookie Theft picture descriptions using graph theory and spatio-semantic graphs. Ours is not the first study to characterize elicited narrative paths on The Cookie Theft description task. Mirheidari et al. used features extracted from speech acoustics and automated transcripts, which characterized the timing on and between areas of interest in the picture, to train an automated classifier to label participants as AD or Healthy Control (19). In contrast, our contribution is a new representation generated from transcripts only, resulting in features that capture the visuospatial path the participant takes as they navigate the content units of the picture without accounting for timing. Further, we individually validate the features on a large-scale dataset that contains a number of subgroups of participants with varying degrees of cognitive status and progression profiles. This allows us to verify that performance patterns coincide with expectations informed by the extant literature on cognition and dementia. A natural extension of our work is to include the timing information as per Mirheidari et al. (19). Other notable studies process participant transcripts from the Cookie Theft Task using natural language processing methodologies to train classifiers to similar ends as Mirheidari et al. (26, 27). These studies use co-occurrence and semantic similarity representations gleaned from transcripts, with content information units and other linguistic features to improve classification of patient transcripts as healthy/control or MCI/AD. While our study encodes CIUs (*via* the graph nodes) and their co-occurrence (*via* the graph edges), we additionally visualize the transcripts and CIUs in the two-dimensional space relative to the Cookie Theft picture itself.

The first analysis revealed differences between the cognitively impaired and cognitively unimpaired groups that may reflect a combination of deficits in visuospatial, attentional, and organizational abilities. With regard to spatial orientation, the Cognitively Impaired group described more of the left side of the picture than the right, in contrast to the Cognitively Unimpaired group (Average *x*). This is notable because the right side actually contains more target CIUs than the left, but its full description requires more abstract inferences (e.g., the mom doesn't notice the children) than does the left side. This finding is corroborated by a study also using data from the DementiaBank database, in which many of the spatial neglect features indicated that the participants with dementia were less perceptive on the right side of the image (28). The Cognitively Impaired group also showed more shifts in attention between nodes on the left and right sides of the picture (higher Standard Deviation of *x*). This attention shifting by the Cognitively Impaired group was also evidenced by the Cross Ratio feature, with a higher number of crossings between quadrants than staying within a quadrant during their description, and more sporadic internode transitions and node repeats than the Cognitively Unimpaired group.

A number of other features seemed to be indicative of poor organization and perhaps memory deficits in the Cognitively Impaired group relative to Cognitively Unimpaired. The Cognitively Impaired group consistently had longer descriptions overall (Total Path Distance), and longer descriptions to reach a similar number of CIUs as the Cognitively Unimpaired group (Total Path Distance/Unique Nodes). This finding aligns with prior work showing reduced density of information and increased use of non-specific words in cognitive decline (6, 29, 30). Similarly, the Cognitively Impaired group's descriptions tended to repeat nodes more frequently, a finding that The Cookie Theft task has revealed before (31). Taken together, these results portray Cognitively Impaired picture descriptions as less organized and less efficiently constructed than those of the Cognitively Unimpaired group. This is in keeping with prior literature.

The second analysis attempted to find features that distinguished performance between the two Cognitively Unimpaired groups, CUS and CUD. As both groups are characterized by normal cognition, any differences that may implicate cognitive decline would be expected to be subtle and difficult to detect. We found that one feature, Total Path Distance/Unique Nodes, approached statistical significance (*p* = 0.0503), with marginal means of 136 for CUS and 142 for CUD. It is of note that this trend is similar to that observed in the comparison between the Cognitively Unimpaired and Cognitively Impaired groups. This overall pattern suggests that spatio-semantic graphs be further explored in preclinical and mild populations for evidence of early cognitive changes. It is likely that larger sample sizes are required to adequately assess the value of these features in these early clinical populations.

It is important to note the limitations in this study. Data collection for this study involves some labor-intensive steps. In this implementation, listeners have manually identified CIUs in the transcripts of spoken participant picture descriptions. Future work will focus on automated extensions utilizing automatic speech recognition algorithms to reduce this workload. Next, population variance is quite heterogeneous between the diagnosis groups across the two sets of ANCOVAs. Only one of the features in the Cognitively Impaired vs. Cognitively Unimpaired models (with the Unique Nodes covariate included) that reached significance, Average *x*, passed Levene's test of homoscedasticity. As the other features violated this assumption of an ANCOVA, the probability of significance of those models may be underestimated (24). The large variance in the Cognitively Impaired diagnosis group may stem from a wider array of causes of clinical impairment, which may be difficult to control for. To resolve this problem in a future study, it is advisable to balance the group sizes by increasing the sample size of the clinical group, as violations of the assumption of homoscedasticity are less important with equal group sizes. Also, because the primary purpose of this study was to evaluate the novel approach and validate its utility for detecting cognitive impairment, there is no evaluation of the marginal value of these features above and beyond other language-based features used in machine learning models of cognitive impairment based on speech (32). Such an evaluation in follow-on work will determine the extent to which these features make unique contributions to the models and improve separability between clinical groups. Finally, we did not identify statistically significant differences between CUS and CUD participants using features derived from the Spatio-Semantic Graphs. We posit that this reflects reduced effect size when detecting very early cognitive decline. However, as many of the features are found to be sensitive to differences in later stages of decline and the group trends approaching significance in Table 2 are in the correct direction, spatio-semantic graphs should be further analyzed in larger scale clinical studies involving participants with very early cognitive decline.

## Data Availability Statement

The raw data supporting the conclusions of this article will be made available by the authors, without undue reservation.

## Ethics Statement

The studies involving human participants were reviewed and approved by the Health Sciences Institutional Review Board of the University of Wisconsin and the other medical centers involved in data collection. The patients/participants provided their written informed consent to participate in this study.

## Author Contributions

PA, RK, VB, JL, and KM contributed to the writing and editing of the manuscript. PA and KB performed statistical analyses. All authors contributed to manuscript revision, read, and approved the submitted version.

## Funding

This research was supported by the National Institute on Deafness and Other Communication Disorders, National Institutes of Health Grant R01DC006859; National Institute on Aging (NIA), NIH Grant R01AG027161; NIA R01AG070940; NIA R01AG054059; NIA AG03705; and NIA AG05133.

## Conflict of Interest

VB and JL are cofounders and have equity in Aural Analytics Inc. The remaining authors declare that the research was conducted in the absence of any commercial or financial relationships that could be construed as a potential of conflict of interest.

## Publisher's Note

All claims expressed in this article are solely those of the authors and do not necessarily represent those of their affiliated organizations, or those of the publisher, the editors and the reviewers. Any product that may be evaluated in this article, or claim that may be made by its manufacturer, is not guaranteed or endorsed by the publisher.
