# Supplementary material for: Spatio-Semantic Graphs From Picture Description: Applications to Detection of Cognitive Impairment
Source: Front Neurol. 2021 Dec 9;12:795374. doi: 10.3389/fneur.2021.795374 (PMC8696356; doi:10.3389/fneur.2021.795374)
Supplement: Supplementary file 1 [file Table_1.DOCX]

**Content Information Units (Semantic Units) Coding Definitions**

| **Unit Number** | **Unit Defined** | **Additional Notes** |
| --- | --- | --- |
| **Subjects** |  |  |
| **1** | Boy (brother, son, junior, young man) | Accept any term that refers to a young boy (e.g. lad, gent, etc.)  Do not accept “guy” or “kid” or a name alone, like "Bobby" |
| **2** | Girl (sister, daughter, gal, young lady) | Accept any term that refers to a young girl.  Do not accept a name alone, like "Sally" |
| **3** | Woman (mother, lady) | Accept any term that refers to an adult woman (e.g., mom) |
| **Places** |  |  |
| **4** | Kitchen |  |
| **5** | Outside (yard, backyard) | Accept any term that refers to something that is outside (e.g., outdoors, trees, etc.) |
| **Objects** |  |  |
| **6** | Cookie | Accept specific cookies (e.g., snickerdoodle cookie, chocolate chip cookie). Do not accept "cookie jar" (this should count only for [+ 7]) |
| **7** | Jar | Accept cookie jar |
| **8** | Stool | Accept only terms related to stool (e.g., three-legged stool).  Do not accept chair, ladder, footstool (Make sure to mark these as semantic errors as well, but do not add [: stool]) |
| **9** | Sink | Do not accept "faucet" |
| **10** | Plate (dish) | Accept any dish related terms (e.g., plate, cup, saucer). |
| **11** | Dishcloth (towel) | Accept any term related to a dish cloth (e.g., dishrag) |
| **12** | Water |  |
| **13** | Window |  |
| **14** | Cupboard (cabinet) |  |
| **15** | Dishes | Accept plurals of dishes (e.g., plates, cups)  Accept within the term (washing the dishes) |
| **16** | Curtains | Accept any term related to curtains (e.g., drapes) |
| **Actions/Facts** |  |  |
| **17** | Boy taking/stealing | Accept any term related to stealing (e.g., robbing, grabbing, sneaking, “getting into the cookie jar”)  Do not accept terms that are not specific to stealing (e.g., getting, reaching) |
| **18** | Boy or stool falling | Accept any term related to falling (e.g., crashing, tipping) |
| **19** | Woman drying/washing plates/dishes | Also accept “doing the dishes” or “wiping dishes” |
| **20** | Water overflowing/spilling | Do accept: "water is running out of the sink"  Do not accept "flooding" |
| **21** | Action performed by the girl | Has to be an action, cannot be a mental state  Accept laughing, stealing, helping, “girl holding her hand to her mouth,” etc.  Do not accept mental states: thinking, emotions, etc. ; do not accept “standing there waiting” or “looks like she wants a cookie” |
| **22** | Woman unconcerned by overflowing | Also accept: “Woman doesn’t notice overflowing.”  *Mom should be agent |
| **23** | Woman indifferent to the children | Also accept: “Woman doesn’t notice children.” “Woman unconcerned with the children.”  Do not accept: "mom has her back to the kids"  *Mom should be agent |

Adapted from Croisile B, Ska B, Brabant MJ, Duchene A, Lepage Y, Aimard G, Trillet M. Comparative study of oral and written picture description in patients with Alzheimer's disease. Brain Lang. 1996 Apr;53(1):1-19. doi: 10.1006/brln.1996.0033. PMID: 8722896, by Cognitive-Communication in Aging and Neurogenic Disorders Lab, PI: KD Mueller (2021).
